# An *in situ* Bi-decorated BiOBr photocatalyst for synchronously treating multiple antibiotics in water†

**DOI:** 10.1039/c8na00197a

**Published:** 2018-12-12

**Authors:** Feng Cao, Jianmin Wang, Yunan Wang, Jun Zhou, Song Li, Gaowu Qin, Weiqiang Fan

**Affiliations:** Key Laboratory for Anisotropy and Texture of Materials (Ministry of Education), School of Material Science and Engineering, Northeastern University Shenyang 110819 China qingw@smm.neu.edu.cn; School of Chemistry & Chemical Engineering, Jiangsu University Zhenjiang 212013 China fwq4993329@yahoo.com

## Abstract

Currently, there is an urgent demand for developing new materials to remove antibiotics in the water environment, especially for the simultaneous degradation of multiple antibiotics. Here, we fabricated a novel Bi/BiOBr heterostructure *via* an *in situ* solvothermal strategy, and it exhibited excellent visible-light-responsive photocatalytic activity for synchronously removing multiple antibiotics coexisting in water. The Bi nanoparticles could extend the light absorption spectra of the sample and further facilitate electron–hole pair separation. The in-depth electron spin resonance (ESR) results confirm that the active species in Bi/BiOBr are holes (h^+^) and superoxide radicals (·O_2_^−^) under irradiation, and it is also proved that Bi could selectively reduce the formation of ·O_2_^−^ in the BiOBr matrix. The coexisting system of TC (tetracycline hydrochloride), CIP (ciprofloxacin) and DOX (doxycycline) could be simultaneously photodegraded to approximately 0% within 30 min by the Bi/BiOBr photocatalyst.

## Abstract 2

A well-defined Bi/BiOBr flowerlike heterostructure has been fabricated *via* an *in situ* solvothermal strategy, and it exhibited excellent photocatalytic activity for synchronously removing multiple antibiotics coexisting in water.

## Graphical abstract

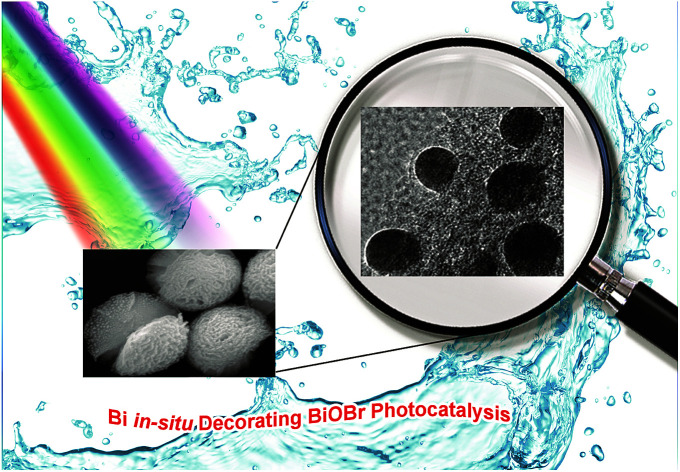

## Introduction

Semiconductor photocatalytic technology can offer the possibility of eliminating toxic chemicals through a green environmental route.^1,2^ Many previous studies have realized the photodegradation of antibiotics in water systems, and several reported photocatalysts present very high degradation efficiencies. For example, Shi *et al.* showed that a nitrogen-doped graphene quantum dot (NGQDs)-BiOI/MnNb_2_O_6_ heterostructure could degrade TC to 0% within 60 min.^3,4^ Most aqueous solutions with antibiotic samples applied for photocatalytic experiments have mainly focused on bare antibiotics; however, multiple antibiotics commonly coexist in real environmental wastewater, which limits the experimental photocatalytic data for practical applications.^5^ To balance the values between theory and practice, it is an important challenge to understand the degradation process of multi-component pollutants. Therefore, exploring suitable photocatalysts and further investigating their potential for synchronously treating multiple antibiotics in water will be important topics in this field.

Bismuth oxyhalides (BiOX, X = Br and I) as typical layered tetragonal semiconductors have been widely applied in the fabrication of visible-light-responsive photocatalysts.^6,7^ However, the photocatalytic performance of bare BiOX (X = Br and I) is still limited because of its high electron–hole recombination rate and poor surface adsorptive ability. Combining BiOX (X = Br and I) with noble metals to obtain uniform heterostructures is an effective strategy to improve the photocatalytic activity. For instance, Yu *et al.* demonstrated that different noble metal particles (Ag, Pt, Pd, *etc.*) could largely improve photocatalytic activities of BiOX (X = Br and I), which could be attributed to the strong visible light absorption and low recombination rate of the e^−^/h^+^ pairs caused by the presence of noble metal particles.^8–10^ However, the introduction of noble metals generates the unavoidable problem of high production costs, and so, replacing noble metals with transition metals will be key to designing photocatalysts with the BiOBr–metal form. Moreover, metallic Bi has been verified to be able to largely improve the photodegradation efficiency of BiOX (X = Cl, Br and I) for various dyes through *in situ* synthesis in many studies, which can effectively decrease the recombination rate of electron–hole pairs and improve their light adsorption as a result of surface plasmon resonance (SPR).^11–18^

In this work, we fabricated a novel visible-light-responsive photocatalyst (Bi/BiOBr), in which Bi nanoparticles were *in situ*-embedded in the BiOBr hierarchical structure with diethylene glycol (DEG) as the reaction medium. Compared to previous studies, the present preparation method relies on *in situ* reduction and growth of Bi nanoparticles on the surface of BiOBr, which can suppress the agglomeration of 0D nanoparticles to expose more active sites. The visible-light photodegradation ability of the Bi/BiOBr nanomaterial was in-depth studied by degrading two-component/three-component mixed antibiotics. The effect of the heterostructure on the photocatalytic performance of Bi/BiOBr hierarchical microflowers has been discussed compared to that of pure BiOBr. Meanwhile, the Bi/BiOBr sample is very air-stable, and its activity remains effective after cyclic experimental tests.

## Experimental

### Preparation of samples

Typically, appropriate amounts of Bi(NO_3_)_3_·4H_2_O and PVP (polyvinylpyrrolidone) were mixed into 10 mL of DEG (HOCH_2_CH_2_OCH_2_CH_2_OH) under magnetic stirring. Then, the mixture was transferred into DEG solution (10 mL) containing KBr. After continuous stirring for 20 minutes, the solution mixture was transferred and sealed in a Teflon-lined autoclave (25 mL). It was kept for 12 h at 180 °C and then cooled naturally. A precipitate was obtained after separation, washing and drying. Meanwhile, bare BiOBr powder was synthesized *via* the same method without DEG.

### Characterization of samples

X-ray diffraction (XRD) was performed using a Rigaku-D/max 2500V using Cu Kα radiation. X-ray photoelectron spectroscopy (XPS) was performed using a Thermo instrument using Al Kα radiation. Scanning electron microscopy (SEM) was performed using a JEOL JEM-7100F. TEM was performed using a JEOL JEM-200CT at 200 kV accelerating voltage. The optical ability was examined using a UV-visible spectrophotometer (PerkinElmer Lambda 750S). ESR was measured using a Bruker model A300-10/12 spectrometer.

### Photocatalytic activity studies

Photocatalytic reactions were performed in a Pyrex reactor using a 300 W Perfect xenon lamp with or without a UV-cut filter (400 nm). 60 mg of photocatalyst was poured into 75 mL fresh pollutant. Prior to irradiation, they were stirred for 60 minutes to ensure that the adsorption equilibrium had been reached. During the photoreactions, 4 mL of reaction solution were taken out at given time intervals for chemical analysis. The dye concentration was analysed by UV-vis absorption spectroscopy. The antibiotic concentration was determined using a Waters e2695 high-performance liquid chromatograph (HPLC). The wavelengths of the UV detector were set at 265 nm (DOX), 280 nm (CIP) and 278 nm (TC).

## Results and discussion

**Fig. 1:**
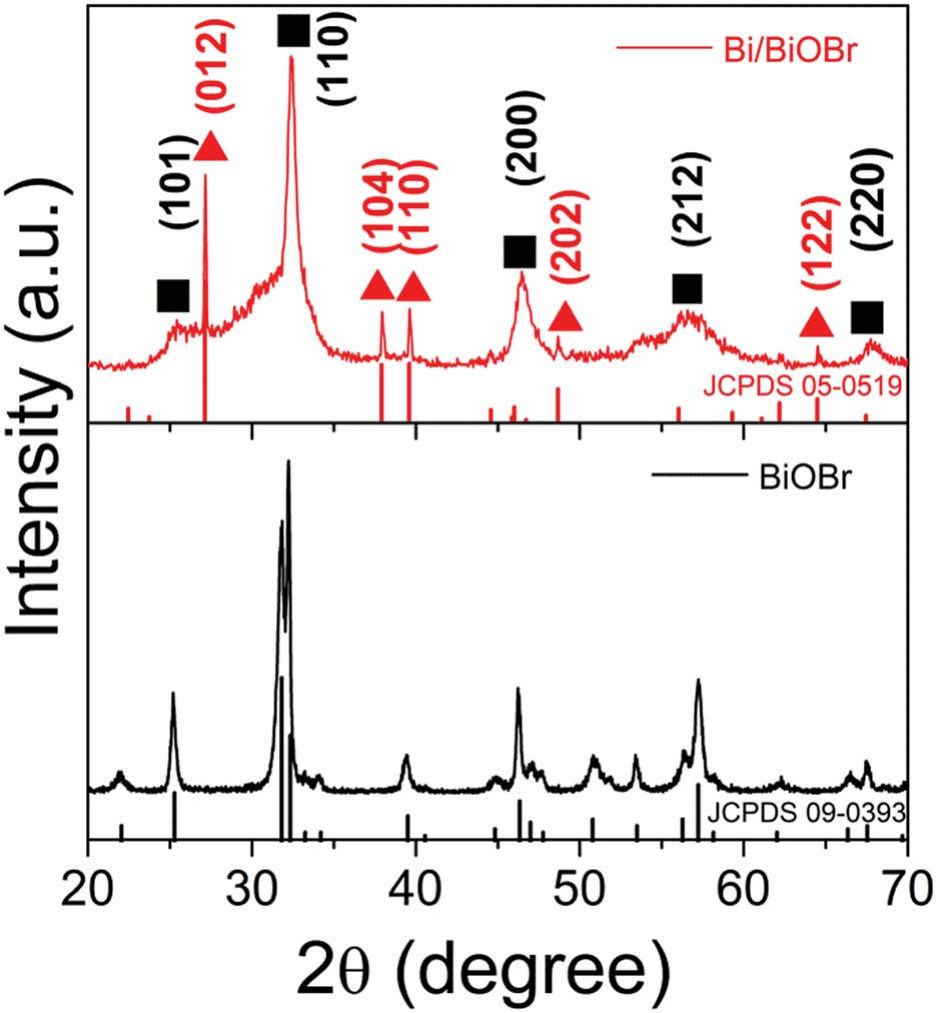
XRD patterns of the Bi/BiOBr and BiOBr samples.

The phase purity and composition were first measured by XRD characterization. As shown in Fig. 1, the observed reflection peaks at 2*θ* = 25.16, 32.20, 46.20, 57.12 and 67.40° correspond to the planes of (101), (110), (200), (212) and (220), in order, which can be readily indexed to the tetragonal phase of BiOBr [space group: *P*4/*nmm* (129)] (corresponding JCPDS File no. 09-0393). At the same time, the diffraction peaks at 2*θ* = 27.17, 38.27, 39.67, 48.93 and 64.68° correspond to the planes of (012), (104), (110), (202) and (122), in order, which can be readily indexed to metallic Bi (JCPDS 05-0519). No impurities can be detected from this pattern, which confirms the successful fabrication of the Bi/BiOBr heterostructure. For comparison, bare tetragonal BiOBr (JCPDS 09-0393) was also prepared by the same method without adding the reducer DEG solvent.

**Fig. 2:**
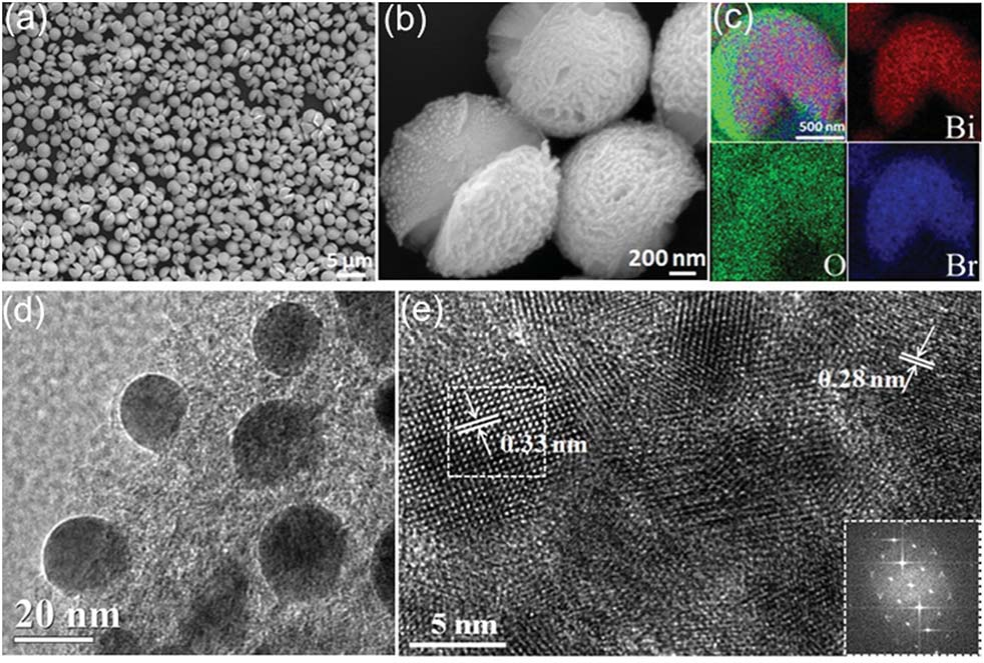
(a) Low-magnification FESEM image of the Bi/BiOBr nanoflowers. (b) Enlarged FESEM image. (c) The corresponding elemental mapping images of Bi, O and Br elements; (d) representative TEM image; and (e) HRTEM image and FFT pattern (inset) of the edge area of the Bi/BiOBr nanoflowers.

The SEM image of the Bi/BiOBr nanoflowers in Fig. 2a shows that they have a uniform flowerlike morphology. The particle size was in the range of 1–3 μm. Fig. 2b further reveals that the microflowers consist of overlapping nanoplatelets and their thickness is approximately 25–35 nm. The corresponding XEDS energy mapping images of the microflowers in Fig. 2c firmly identify their composition, in which Bi, O and Br have been detected. Furthermore, the SEM image of the BiOBr sample displays a nanoflake morphology (Fig. S1†). The TEM image in Fig. 2d further shows that many Bi nanoparticles with approximately 15 nm diameter were evenly attached to the BiOBr surface. Two types of lattice fringes were observed in the HRTEM image (Fig. 2e), and they correspond to the (102) planes of BiOBr and (012) planes of Bi, respectively.

**Fig. 3:**
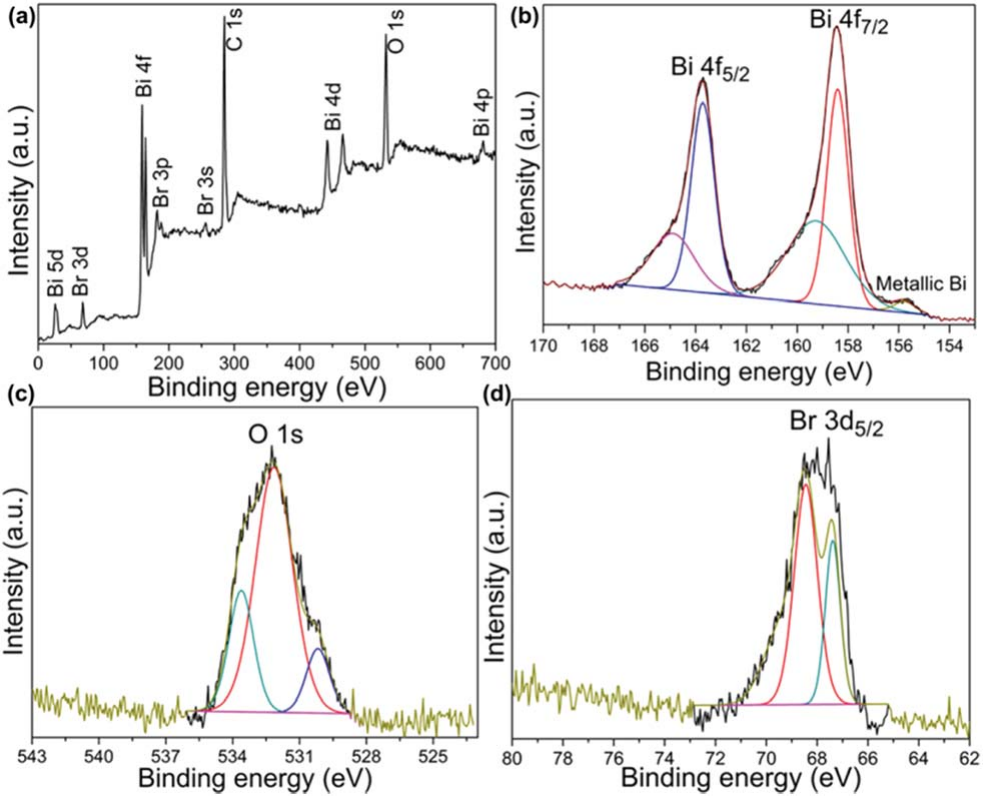
XPS spectra of the Bi/BiOBr nanoflowers: (a) survey; (b) Bi 4f; (c) O 1s; (d) Br 3d.

XPS measurements were also performed to investigate the chemical composition and elemental valence states.^19^ The survey XPS result (Fig. 3a) supports the presence of O, Br and Bi. The Bi 4f XPS spectrum is depicted in Fig. 3b, and it can be split into two parts, Bi 4f7/2 and Bi 4f5/2, and the shake-up satellite peak at 155.7 eV was ascribed to metallic Bi.^20,21^ In the O 1s spectra of Fig. 3c, the peaks at 529.9 eV and 531.5 eV can be ascribed to Bi–O bonds and Br–O bonds in BiOBr.^22,23^ The peaks at 533.0 eV were caused by the surface adsorbed OH group.^24^ In the Br 3d spectra (Fig. 3d), the binding energies of 67.5 eV and 68.5 eV were attributed to Br 3d5/2 and 3d3/2, respectively, which could be assigned to Br in the monovalent oxidation state.^25^

**Fig. 4:**
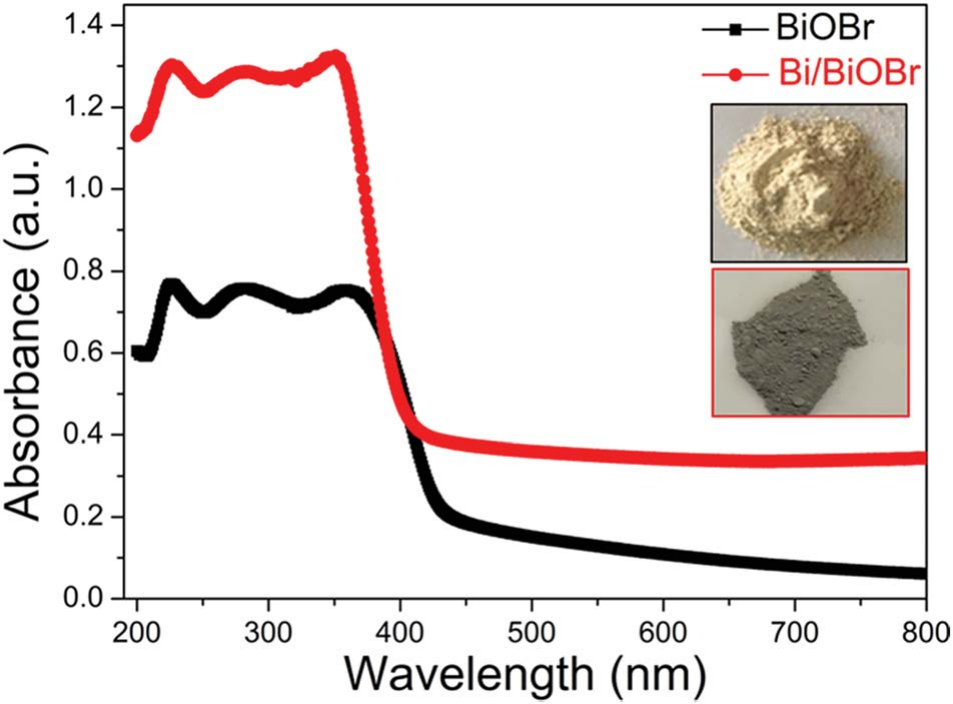
UV-vis solid absorption spectra of Bi/BiOBr and BiOBr samples.

The optical absorption properties of BiOBr and Bi/BiOBr were investigated by UV-vis spectroscopy (Fig. 4). The typical visible light absorption was observed in each sample, which mainly corresponded to the intrinsic narrow band-gap energy of BiOBr.^26^ It can be observed that the absorption ability of the Bi/BiOBr heterostructure has been greatly enhanced compared to that of bare BiOBr, which can be attributed to SPR as a result of the appearance of metallic Bi.^27–29^ The colour change from milky white for the BiOBr sample to dark grey for the Bi/BiOBr sample also suggests that the Bi/BiOBr sample could utilize more solar light. Furthermore, the specific surface area and porous nature of Bi/BiOBr and BiOBr were measured. As shown in Fig. S2† and Table 1, the BET specific surface area of Bi/BiOBr is approximately 20.62 m^2^ g^−1^ and was 3 times higher than that of BiOBr (6.69 m^2^ g^−1^), which is favourable for its use as a photocatalyst.

**Table 1:** Specific surface area and pore structure of the samples

Sample	Specific surface area (m^2^ g^−1^)	Pore size (nm)	Pore volume (cm^3^ g^−1^)
BiOBr	6.69	22.04	0.03
Bi/BiOBr	20.62	8.53	0.05

When this preparation method was extended to BiOCl and BiOI, similar morphology was obtained (Fig. S3 and S4†), but the *in situ* decoration of Bi on BiOCl and BiOI failed (Fig. S5†). Therefore, it is important to understand how the Bi selectively forms in the BiOBr system. Considering the stability constants of BiOCl (*K*_sp_ = 1.8 × 10^−31^), BiOBr (*K*_sp_ = 3 × 10^−7^) and BiOI (*K*_sp_ = 3 × 10^−17^), the Bi^3+^ ions will more easily dissociate in BiOBr, which benefits the reduction of Bi^3+^ to Bi.^30–32^ Moreover, DEG plays a critical role in the nucleation or growth control of BiOX (X = Cl, Br and I) hierarchical microflowers in this experiment. In detail, the roles of DEG can be divided into three parts: (1) solvent; (2) “soft” template and/or capping agent;^33,34^ and (3) reducing agent.^35,36^ DEG can coordinate with Bi^3+^ to form a homogeneous precursor prior to decomposition, which is similar to ZnSe microflowers and Ni_7_S_6_ microflowers. Owing to the large stability constant, Bi^3+^ was partially reduced to Bi by DEG in the case of the BiOBr system.

**Fig. 5:**
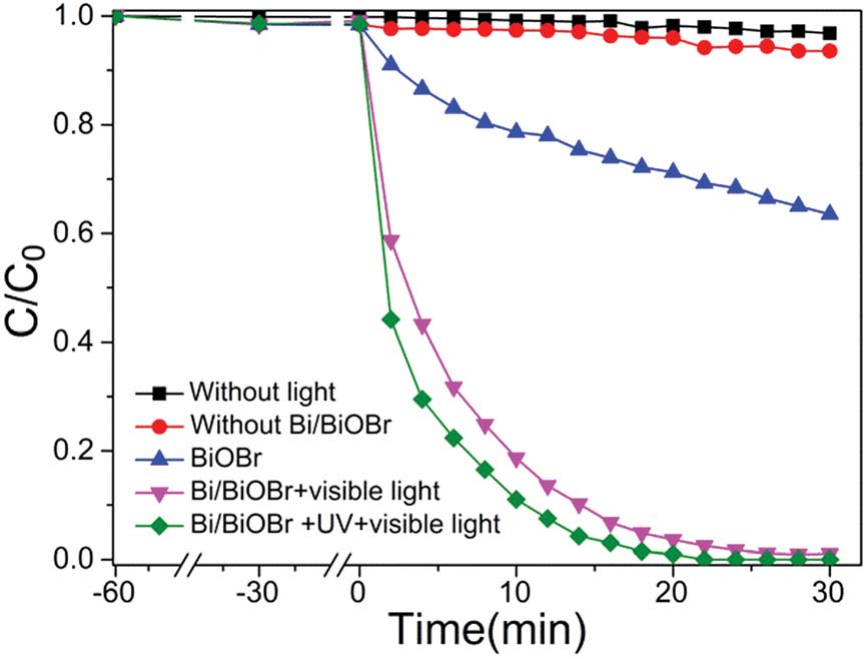
Photocatalytic activities for degradation of TC solution under visible-light irradiation at room temperature in the presence of Bi/BiOBr and BiOBr samples.

Recently, much attention has been paid to antibiotic photodegradation under visible light irradiation. In the present work, we selected TC as the target compound to evaluate the photodegradation activity of the Bi/BiOBr heterostructure. Fig. 5 shows the relationship of TC concentration variations with irradiation times for Bi/BiOBr and BiOBr samples. The photodegradation of antibiotics by the Bi/BiOBr nanomaterial is the combined effect of adsorption, photolysis and photocatalysis.^37^ Control experiments of the adsorption and photolysis effect demonstrate negligible photoactivities, suggesting that the reaction is mainly driven by a photocatalytic process. Bi/BiOBr exhibits an extraordinarily high antibiotic photodegradation efficiency (almost 100% TC removed within 20 min), which is obviously higher than that of the BiOBr photocatalyst (30% TC removed within 40 min). In addition, the effect of the filter cut-off (*λ* ≤ 400 nm) on the antibiotic photodegradation was investigated, and high antibiotic photodegradation efficiency under UV and visible light irradiation was observed. For comparison, the photocatalytic performance of the BiOI nanomaterial was also investigated, and the decomposition of TC proceeded much slower than for the Bi/BiOBr sample even though they have very similar morphologies (Fig. S6†). The photodegradation of DOX and CIP antibiotics was also studied. DOX and CIP could be completely degraded after 20 min of irradiation (Fig. S7†). Meanwhile, photodegradation with the BiOBr sample was relatively slow, *i.e.*, 28% of DOX or CIP can be removed after 40 min of irradiation. In summary, compared to previously reported literature, the Bi/BiOBr microflowers exhibited a superior photodegradation antibiotic ability.^38^

**Fig. 6:**
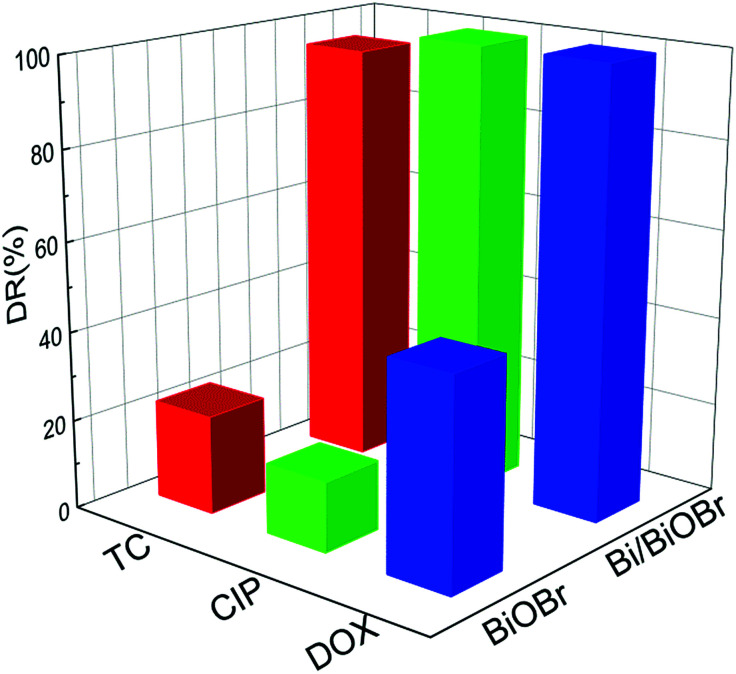
The photocatalytic degradation of ternary mixed antibiotic TC–DOX–CIP solution with 20 min irradiation time for Bi/BiOBr and BiOBr samples.

It is common knowledge that several kinds of antibiotics are present in real pollutant wastewater. Therefore, efficient and simultaneous removal of multiple antibiotics is urgently needed. The simultaneous photodegradation of two-component/three-component antibiotics was studied. In the case of two-component antibiotic photodegradation (Fig. S8†), Bi/BiOBr nanoflowers exhibited much higher photocatalytic activities than BiOBr nanoplates. Emphatically, the excellent photodegradation performance was also retained in three-component antibiotic degradation experiments. Fig. 6 shows the relationship of DOX, CIP and TC concentration variations with irradiation time for Bi/BiOBr and BiOBr sample nanomaterials. It was clearly observed that for the TC/CIP/DOX three-component antibiotics, the Bi/BiOBr photocatalyst also displayed a significantly higher photocatalytic activity than the BiOBr photocatalyst. After 20 min of irradiation, 99% of CIP or DOX and 88% of TC can be degraded in the presence of the Bi/BiOBr photocatalyst. Additionally, all TC/CIP/DOX mixed antibiotics were decomposed within 30 minutes, and the degradation rate of the multiple-component antibiotics was slower than that of the single-antibiotic solution.

**Fig. 7:**
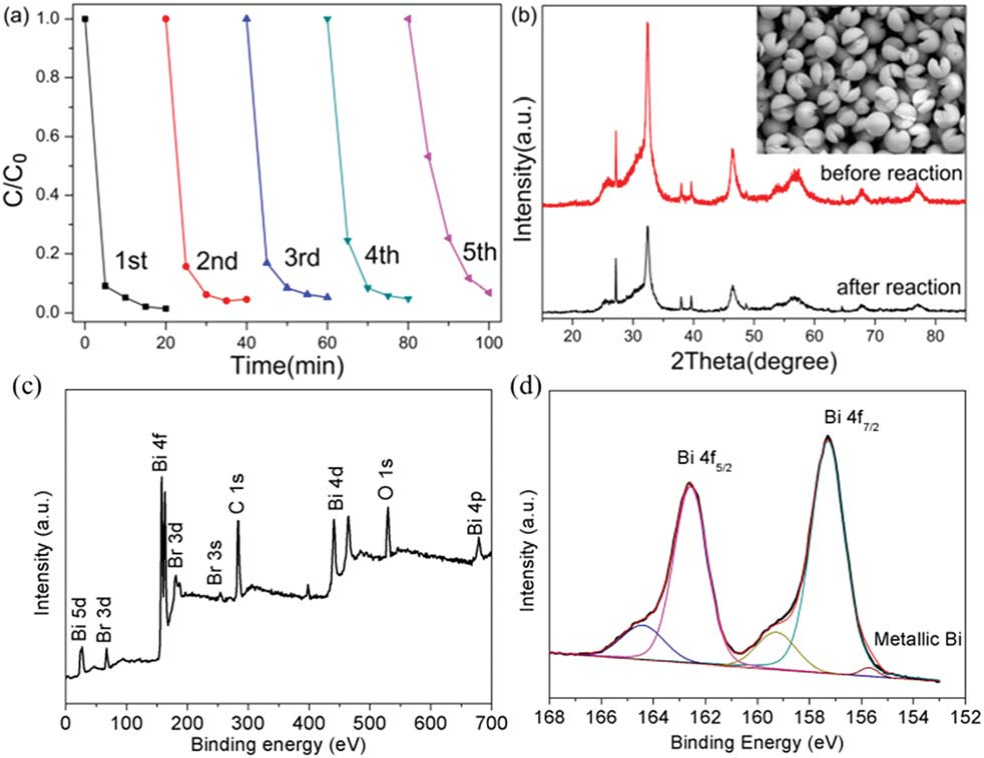
(a) Reusability of Bi/BiOBr microflowers for photodegradation of TC; (b) XRD pattern and SEM result before and after 5 reusability tests; XPS spectra of the Bi/BiOBr after 5 reusability tests: (c) survey; (d) Bi 4f.

To check the stability of the photocatalytic process, we carried out a photocatalytic experiment under prolonged light illumination. We can find in Fig. 7 that the photocatalytic activity can be retained without a noticeable decrease after five cycles, demonstrating the excellent stability of the Bi/BiOBr nanomaterial. Additionally, the SEM and XRD patterns of Bi/BiOBr were investigated for 5 reusability runs in TC photodegradation. There is no obvious change in the photocatalytic reaction. Moreover, XPS results display the peak at about 155.7 eV that is assigned to metallic Bi, which further confirms the existence of metallic Bi after photocatalytic reaction. These results suggest that our Bi/BiOBr nanomaterial is an efficient and stable visible light photocatalyst.

**Fig. 8:**
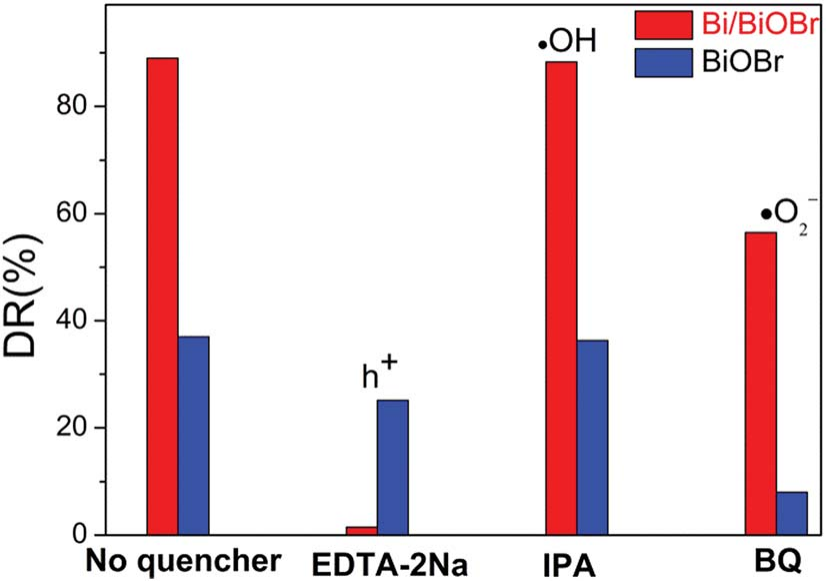
Trapping experiment of active species during the photocatalytic reaction with visible light irradiation: EDTA-2Na as a scavenger for holes (h^+^); IPA (iso-propanol) as a scavenger for hydroxyl radicals (·OH); BQ (1,4-benzoquinone) as a scavenger for superoxide radicals (·O_2_^−^).

**Fig. 9:**
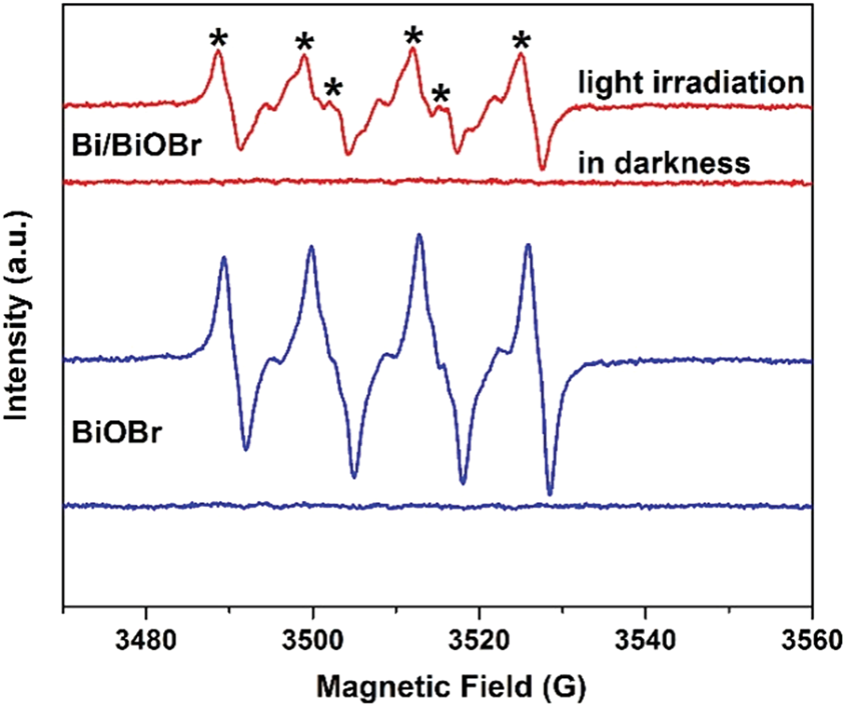
ESR spectra of the as-synthesized Bi/BiOBr and BiOBr samples for DMPO–·O_2_^−^.

In order to confirm the main active radical species and the potential reaction mechanism for superior photodegradability of the Bi/BiOBr nanomaterial, trapping experiments of active species were performed *via* photocatalytic reaction (Fig. 8). The degradation rate (DR) decreased in the presence of superoxide radical scavenger BQ and hole scavenger EDTA-2Na, revealing that ·O_2_^−^ and h^+^ are both active species for TC degradation, and the holes h^+^ play the dominant role in the Bi/BiOBr system, while ·O_2_^−^ plays a stronger role than h^+^ in the presence of the BiOBr sample. However, the CB of BiOBr is positive relative to the potential of O_2_/·O_2_^−^ (−0.33 V *versus* NHE), so CB electrons are unable to easily produce ·O_2_^−^.^39^ However, BiOBr reacts with O_2_ under irradiation, and a similar phenomenon was reported in previous work.^40^ Because the electrons excited into the CB of BiOBr would induce a Fermi level up-shift and benefit the reduction of O_2_, the decoration of Bi significantly reduces the process of generating ·O_2_^−^, which demonstrates that Bi as a co-catalyst is inert to the reaction of O_2_/·O_2_^−^. Due to the position difference between BiOBr's CB (0.15 eV) and the Bi Fermi level (−0.17 eV), photogenerated electrons migrate easily from Bi to the CB of BiOBr; therefore, the heterostructure favours the separation process of charges and promotes the activity of h^+^ for degradation. The above deduction can be well supported by the ESR analysis (Fig. 9). Obvious DMPO–·O_2_^−^ characteristic peaks appeared after light irradiation, which further indicates the generation of ·O_2_^−^ during the degradation of TC, but the signal density was weaker in the presence of Bi/BiOBr because of the decreased amount of ·O_2_^−^, which agrees well with the trapping experiment. The joint action of ·O_2_^−^ and h^+^ leads to the enhanced photodegradation performance of Bi/BiOBr.

## Conclusions

In summary, a novel visible-light-responsive photocatalytic Bi/BiOBr heterostructure has been prepared *via* a simple *in situ* solvothermal synthetic route. The Bi/BiOBr photocatalyst shows excellent visible light photocatalytic properties for antibiotic degradation, compared to bare BiOBr. In two-component/three-component antibiotic solutions, almost 100% of the antibiotics were degraded within 30 min of irradiation. This high photocatalytic activity originates from its hierarchical microflowers. And the photocatalytic activity and structure were retained after 5 cycles. This work demonstrated a powerful strategy for the *in situ* reduction and confined growth of well-defined 3D heterostructures, and this promising performance shows great potential for these materials as photocatalysts for the visible light removal of multiple antibiotics in practical application.

## Conflicts of interest

There are no conflicts to declare.

## Supplementary Material

NA-001-C8NA00197A-s001
