# Supplementary material for: An in situ Bi-decorated BiOBr photocatalyst for synchronously treating multiple antibiotics in water
Source: Nanoscale Adv. 2018 Dec 12;1(3):1124–9. doi: 10.1039/c8na00197a (PMC9473170; doi:10.1039/c8na00197a)
Supplement: NA-001-C8NA00197A-s001 [file NA-001-C8NA00197A-s001.pdf]

Electronic Supplementary Material (ESI) for Nanoscale Advances.
This journal is © The Royal Society of Chemistry 2018

Electronic Supporting Information

In-situ Bi-decorated BiOBr Photocatalyst for Synchronously Treating Multiple Antibiotics in Water

Feng Cao,[a] Jianmin Wang,[a] Yunan Wang,[a] Jun Zhou,[a] Song Li,[a] Gaowu Qin*[a] and Weiqiang Fan*[b]

[a] *Key Laboratory for Anisotropy and Texture of Materials (Ministry of Education), School of Material Science and Engineering, Northeastern University, Shenyang 110819, China.*

[b] *School of Chemistry & Chemical Engineering, Jiangsu University, Zhenjiang 212013, China.*

**Corresponding author: qingw@smm.neu.edu.cn (G. W. Qin) and fwq4993329@yahoo.com (W. Q. Fan).*

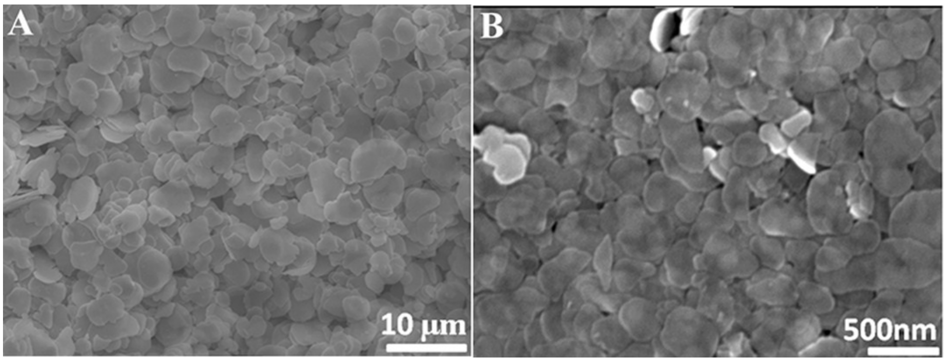

Fig. S1 FESEM images of the BiOBr prepared by a hydrothermal method without the assistant of reducer DEG.

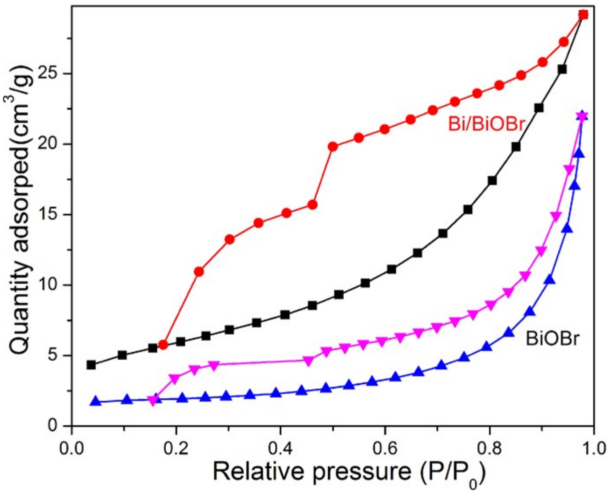

Fig. S2 Nitrogen adsorption–desorption isotherms of Bi/BiOBr and BiOBr samples.

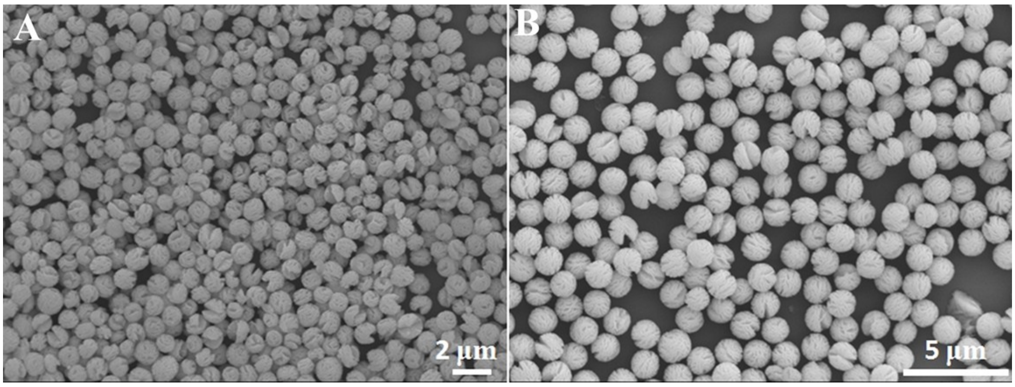

Fig. S3 FESEM images of BiOCl synthesized under the similar conditions unless using KCl instead of KBr.

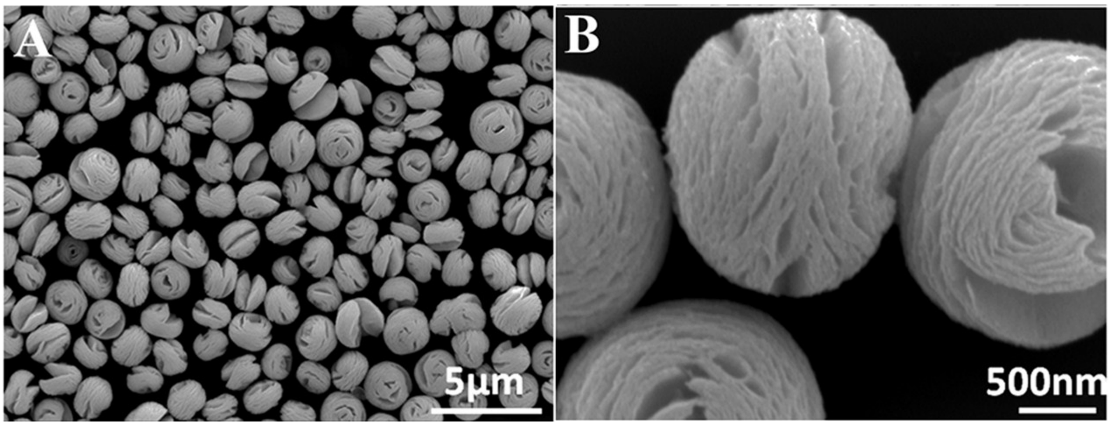

Fig. S4 FESEM images of BiOI synthesized under the similar conditions unless using KI instead of KBr.

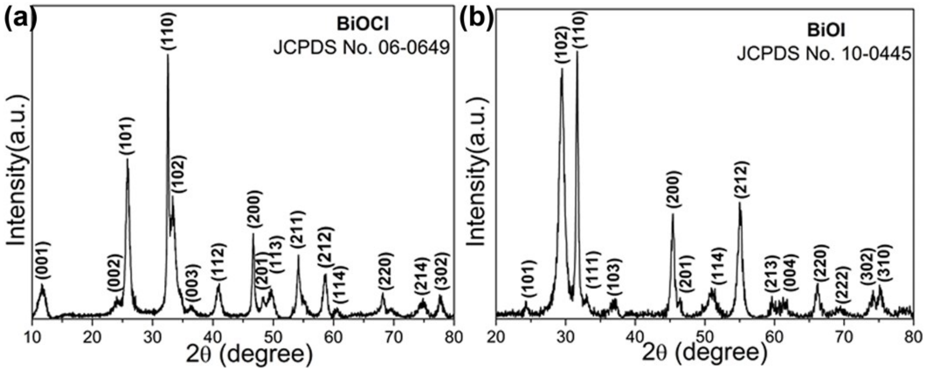

Fig. S5 XRD patterns of products (a) BiOCl and (b) BiOI synthesized under the similar conditions unless using KCl or KI instead of KBr.

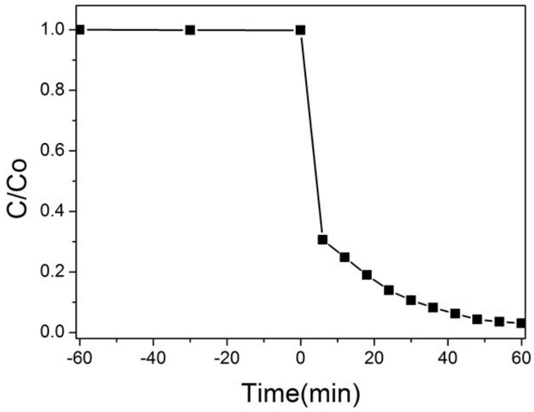

Fig. S6 Photodegradation of TC under visible-light irradiation at room temperature in the presence of BiOI hierarchical microflower.

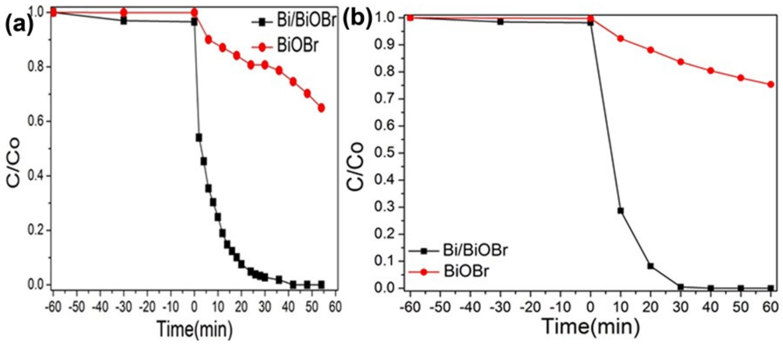

Fig. S7 Photodegradation of (a) DOX and (b) CIP under visible light irradiation at room temperature in the presence of Bi/BiOBr and BiOBr samples.

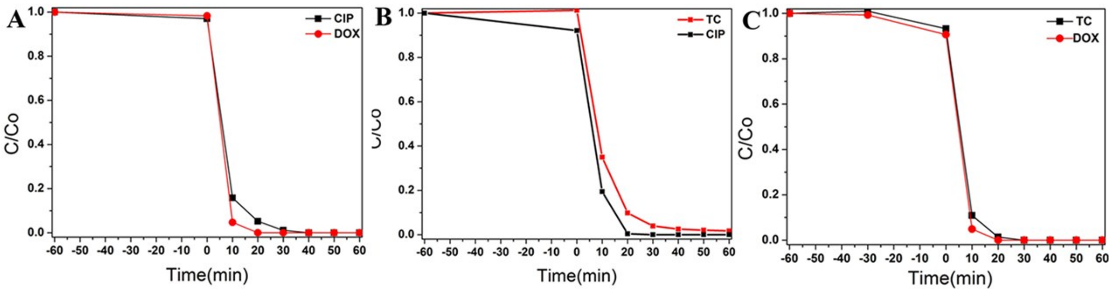

Fig. S8 Photodegradation of two-component antibiotic under visible light irradiation at room temperature in the presence of Bi/BiOBr hierarchical microflowers.
